# Trends in hospital and intensive care admissions in the Netherlands attributable to the very elderly in an ageing population

**DOI:** 10.1186/s13054-015-1061-z

**Published:** 2015-09-30

**Authors:** Lenneke E. M. Haas, Attila Karakus, Rebecca Holman, Sezgin Cihangir, Auke C. Reidinga, Nicolette F. de Keizer

**Affiliations:** Department of Intensive Care Medicine, Diakonessenhuis, PO box 80250, 3508, TG Utrecht, The Netherlands; NICE foundation, Amsterdam, The Netherlands; Amsterdam Medical Centre, Department of Medical Informatics, Amsterdam, The Netherlands; Dutch Hospital Data, Utrecht, The Netherlands; Department of Intensive Care, Martini Hospital, Groningen, The Netherlands

## Abstract

**Introduction:**

The Dutch population is ageing and it is unknown how this is affecting trends in the percentage of hospital and intensive care unit (ICU) admissions attributable to patients aged 80 years or older, the very elderly.

**Methods:**

We present data on the percentage of the very elderly in the general population and the percentage of hospital admissions attributable to the very elderly. We subsequently performed a longitudinal cross-sectional study on ICU admissions from hospitals participating in the National Intensive Care Evaluation registry for the period 2005 to 2014. We modeled the percentage of adult ICU admissions and treatment days attributable to the very elderly separately for ICU admissions following cardiac surgery and other reasons.

**Results:**

The percentage of Dutch adults aged 80 years and older, increased from 4.5 % in 2005 to 5.4 % in 2014 (p-value < 0.0001) and with this ageing of the population, the percentage of hospital admissions attributable to very elderly increased from 9.0 % in 2005 to 10.6 % in 2014 (p-value < 0.0001). The percentage of ICU admissions following cardiac surgery attributable to the very elderly increased from 6.7 % in 2005 to 11.0 % in 2014 in nine hospitals (p-value < 0.0001), while the percentage of treatment days attributable to this group rose from 8.6 % in 2005 to 11.7 % in 2014 (p-value = 0.0157). In contrast, the percentage of very elderly patients admitted to the ICU for other reasons than following cardiac surgery remained stable at 13.8 % between 2005 and 2014 in 33 hospitals (p-value = 0.1315). The number of treatment days attributable to the very elderly rose from 11,810 in 2005 to 15,234 in 2014 (p-value = 0.0002), but the percentage of ICU treatment days attributable to this group remained stable at 12.0 % (p-value = 0.1429).

**Conclusions:**

As in many European countries the Dutch population is ageing and the percentage of hospital admissions attributable to the very elderly rose between 2005 and 2014. However, the percentage of ICU admissions and treatment days attributable to very elderly remained stable. The percentage of ICU admissions following cardiac surgery attributable to this group increased between 2005 and 2014.

**Electronic supplementary material:**

The online version of this article (doi:10.1186/s13054-015-1061-z) contains supplementary material, which is available to authorized users.

## Introduction

In the Netherlands, median age is rising [1] corresponding to the demographic changes observed throughout Europe [2]. Life expectancy in the Netherlands was 70 years for men and 79 years for women in 1950. These figures rose to 79 and 83 years in 2013 and are projected to increase to 84 and 87 years by 2055 [2, 3]. The percentage of people in the Netherlands aged 80 years or older, the very elderly, increased from 1 % in 1950 to 4 % in 2013 and is expected to rise to 9 % in 2040 and 11 % in 2055 [4]. The observed and projected increases in the percentage of the very elderly are consequences of decreased fertility in recent decades, increased life expectancy and high birth rates in the period 1945 to 1955 [2, 3].

Although older patients comprise a minority of the population, they are responsible for a substantial proportion of hospitalizations and healthcare costs, including intensive care unit (ICU) treatment days [5–7]. An increase in the percentage of elderly patients in the general population may profoundly affect utilization of ICU resources [8–11]. Dutch [12] and international researchers [13, 14] have predicted that aging may lead to substantial increases in the demand for ICU treatment. Recently conducted studies show that the percentage of ICU admissions attributable to the very elderly is 13 % for Australia and New Zealand [15], 8.9 % for Finland [16], 12.4 % [17] and 18.2 % [18] for France, 19.2 % for Italy [19], 15.5 % for Norway [20] and 11 % for Spain [21]. In Denmark, this percentage increased marginally from 11.7 % in 2005 to 13.8 % in 2011 [22]. However, it is unclear how the results of these studies relate to the situation in the Netherlands.

A study conducted in the Netherlands between 1997 and 2002 showed that 6.9 % of the ICU patients were very elderly [23]. However, it is unclear what percentage of adult ICU admissions in the Netherlands is currently attributable to very elderly patients and whether this percentage has increased in recent years. Multiple factors, including how the healthcare system is organized, ICU admission criteria and ethical choices, may influence decisions on whether very elderly patients are admitted to the ICU [24]. In this study, we describe trends in the percentage of the Dutch population aged 80 years or older and the percentage of hospital and ICU admissions attributable to the very elderly. We also examine trends in the acute physiology and chronic health evaluation II (APACHE II) [30] and simplified acute physiology score II (SAPS II) [31] predicted probabilities of mortality and length of ICU stay for the very elderly and the proportion of very elderly patients with chronic renal, cardiovascular or immunological insufficiency, a malignancy and who were admitted to the ICU for medical reasons. Although some of these trends have previously been compared in general, hospital and ICU populations in Denmark [22], it is unclear whether these results are applicable to other countries, in general, or the Netherlands, in particular.

## Methods

### Data sources

We used data from Statistics Netherlands, the Dutch national statistical service, on the number and percentage of adults aged 80 years or older in the Dutch population as a whole on 1 January [25] of each year from 2005 to 2014. We also used data on the number and percentage of hospital admissions attributable to the very elderly in the period 2005 to 2014 from the Dutch Hospital Data foundation [26]. The Dutch Hospital Data foundation was founded by the Netherlands Association of Hospitals and the Netherlands Federation of University Medical Centers to manage, maintain and monitor collections of hospital data and to provide information on hospital care. We extracted data from the national hospital care basic registration. This is a registry of hospital admissions and includes demographic patient information, primary and secondary diagnoses in terms of International Classification of Diseases codes, operations performed and other information on treatment. All Dutch Hospitals have provided this information since 1963.

In addition, we used data from the Dutch National Intensive Care Evaluation (NICE) registry [27], a voluntary quality registry that contains all consecutive ICU admissions to participating hospitals. The NICE registry was set up in 1996 to enable hospitals to compare and improve the quality of care in Dutch ICUs [28]. The number of hospitals participating rose from 6 in 1997 to 85 of the 90 Dutch ICUs in 2014 [29]. Participating hospitals deliver demographic, physiological and diagnostic data and the outcomes of all admissions to their ICUs. These data enable the calculation of the APACHE II [30] and SAPS II [31] predicted probability of mortality. The NICE registry is registered according to the Dutch Personal Data Protection Act. The medical ethics committee of the Academic Medical Center stated that medical ethics approval for this study was not required under Dutch national law (registration number W15-160).

#### Definitions and inclusion criteria for data from the NICE registry

We classified an admission as attributable to the very elderly if the patient was aged at least 80 years on admission to the ICU and as being related to cardiac surgery if the APACHE II or IV reason for admission was related to planned or emergency cardiac surgery, as detailed in Additional file 1 [32]. We present data on admissions following cardiac surgery and admissions for other reasons separately. This is because patients admitted to the ICU following cardiac surgery tend to be younger [33], cardiac surgery is only performed in a limited number of hospitals in the Netherlands and the percentage of cardiac procedures performed on the very elderly in the Netherlands has risen since 2005 [34]. An admission was defined as a medical admission if the patient did not come directly from an operating theatre to the ICU. To describe comorbidities we defined a patient as having chronic renal insufficiency if he or she had a chronically raised serum creatinine (above 177 umol/L or 2.0 mg/dL) or had received hemodialysis or peritoneal dialysis for a substantial period before the start of the hospital admission. We defined a patient as having chronic cardiovascular insufficiency if he or she had New York Heart Association class IV heart failure. We defined a patient as having a malignancy if he or she had solid tumor metastases or malignant lymphoma, acute leukemia or multiple myeloma. We defined a patient as having immunological insufficiency if he or she used long-term immunosuppressive therapy, or corticosteroid therapy, chemotherapy, radiotherapy in the last year, or had had chemotherapy or radiotherapy for Hodgkins or non-Hodgkins lymphoma at any point before ICU admission, or had documented cell deficiencies. We calculated the APACHE II [30] and SAPS II predicted probability of mortality [31] using standard methods. We defined the ICU length of stay as the number of fractional days between ICU admission and ICU discharge. We analyzed trends in ICU length of stay by classifying the length of each admission as being longer or shorter than the median length of stay over all admissions in all years to all ICUs included in this study. We described the burden of the very elderly on ICUs by examining the number and percentage of admissions and ICU treatment days attributable to them. We obtained the total number of ICU treatment days and the number attributable to the very elderly by summing the length of individual admissions in fractional days in each calendar year in each hospital.

We extracted data from the NICE registry on ICU admissions attributable to patients known to be male or female, aged at least 18 years on admission to the ICU and admitted as a result of medical reasons, planned surgery or emergency surgery between 1 January 2005 and 31 December 2014. We excluded admissions to hospitals with fewer than 10 admissions for other reasons attributable to the very elderly or 10 admissions attributable to younger patients in any calendar year between 2005 and 2014 to increase the stability of estimates of parameters in the logistic models for the percentage of admissions attributable to the very elderly [35]. For the analyses of trends in the predicted probability of mortality and percentage with chronic conditions among the very elderly patients admitted to ICUs, we also excluded all admissions not fulfilling the inclusion criteria for both the APACHE II and SAPS II models for predicting the probability of mortality.

### Statistical analysis

We analyzed changes over time in the percentage of very elderly adults in the Statistics Netherlands and hospital admissions attributable to the very elderly in the Dutch Hospital Data using a generalized linear model, with a constant and linear term for time and a binomial link function. We analyzed changes over time in the absolute number of admissions attributable to the very elderly using generalized linear mixed-effects models with a Poisson link function. We analyzed the proportions of admissions attributable to the very elderly and of the very elderly admitted to an ICU for medical reasons, with each of the chronic conditions and with an ICU length of stay longer than the overall median using generalized linear mixed-effects models with a binomial link function. We analyzed the logarithm of the total number of treatment days attributable to the very elderly, logarithm of the hospital median length of ICU stay and the logit transformed APACHE II and SAPS II predicted probabilities of mortality using linear mixed-effects models. We defined time as the number of whole years since 2005 and included random intercepts and linear terms for time for each hospital in all mixed-effects models.

We performed all analyses using version 3.1.0 of the statistical platform R [37] and estimated the parameters for the generalized linear models using the function glm and for the mixed-effects models using the function glmer in the package lme4 [38]. We considered *p* values smaller than 0.05 as statistically significant and made no corrections for multiple testing. We obtained 95 % confidence intervals using the Wilson score method [39] implemented in the PropCIs package [40].

## Results

We present Statistics Netherlands population data on the number and percentage of Dutch adults, who are very elderly, and Dutch Hospital Data on the number and percentage of adult hospital admissions attributable to the very elderly between 2005 and 2014 in Table 1. These data show that the percentage of very elderly adults in the Netherlands increased from 4.5 % (95 % CI 4.5 % to 4.5 %) in 2005 to 5.4 % (95 % CI 5.3 % to 5.4 %, *p* value <0.0001) in 2014 and that the percentage of hospital admissions attributable to the very elderly increased from 9.0 % (95 % CI 9.0 % to 9.1 %) in 2005 to 10.6 % (95 % CI 10.6 % to 10.6 %) in 2014 (*p* value <0.0001).

**Table 1:** Statistics Netherlands population data and Dutch Hospital Data on the number and percentage of adult hospital admissions attributable to the very elderly for the years 2005 to 2014

	Statistics Netherlands population data		Dutch Hospital Data on hospital admissions	
Year	The very elderly	All adults	The very elderly	All adults
2005	573,573 (4.5 %)	12,707,935	284,484 (9.0 %)	3,145,476
2006	587,016 (4.6 %)	12,752,453	307,986 (9.3 %)	3,316,256
2007	600,842 (4.7 %)	12,793,540	330,360 (9.5 %)	3,491,052
2008	615,489 (4.8 %)	12,859,287	355,635 (9.7 %)	3,675,625
2009	631,208 (4.9 %)	12,957,546	381,782 (9.8 %)	3,881,713
2010	647,994 (5.0 %)	13,060,511	413,450 (10.1 %)	4,079,607
2011	667,547 (5.1 %)	13,153,716	446,385 (10.4 %)	4,289,547
2012	686,015 (5.2 %)	13,243,578	457,074 (10.5 %)	4,341,407
2013	702,820 (5.3 %)	13,316,082	418,457 (10.7 %)	3,924,802
2014	717,089 (5.4 %)	13,386,487	363,630 (10.6 %)	3,437,061

**Fig. 1:**
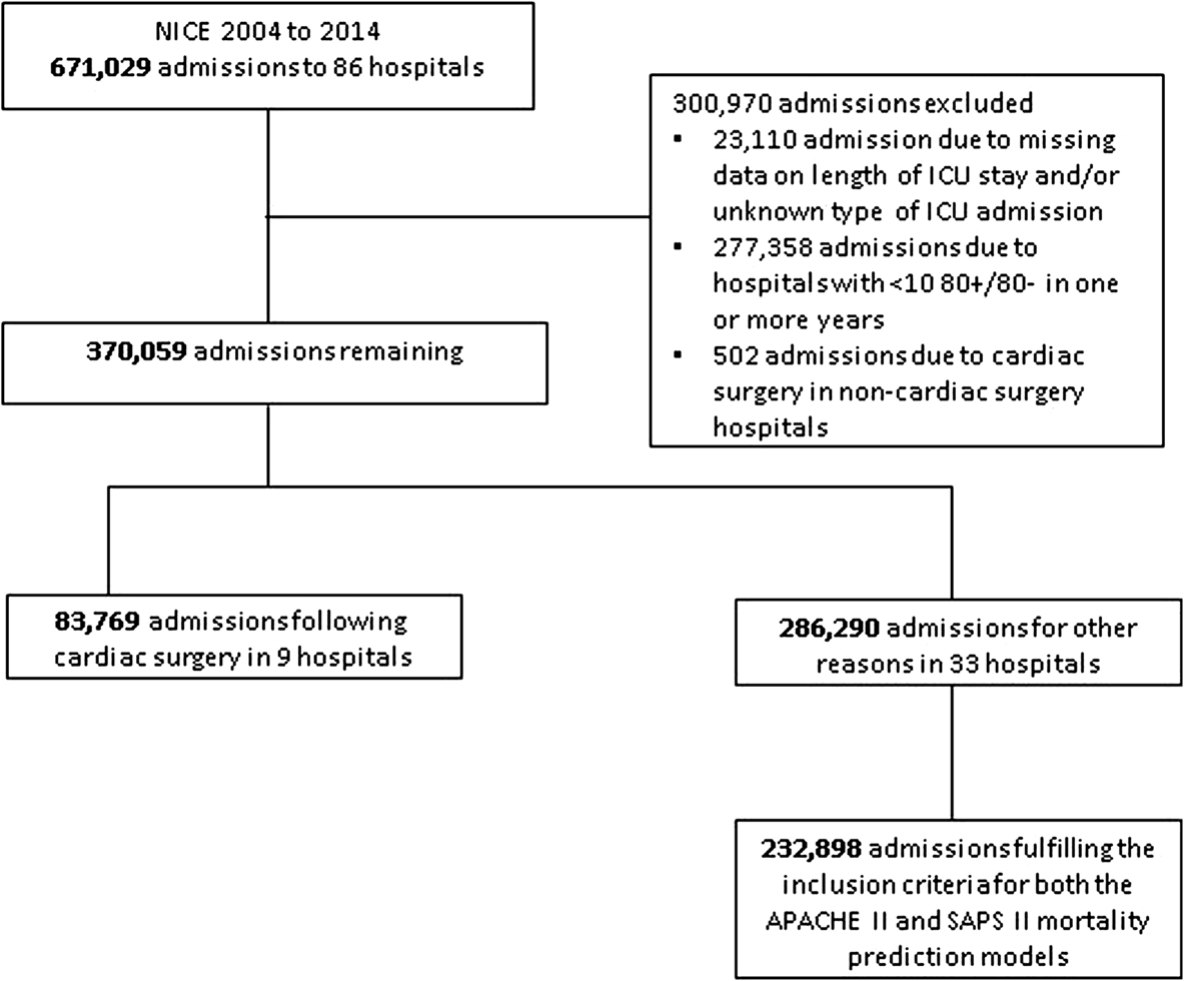
Flow diagram showing the number of intensive care admissions and hospitals included in the analysis. *NICE* Dutch National Intensive Care Evaluation, *APACHE* acute physiology and chronic health evaluation, *SAPS* simplified acute physiology score

We included 83,769 ICU admissions following cardiac surgery to 9 hospitals and 286,290 ICU admissions for other reasons to 33 hospitals (Fig. 1). The 9 hospitals delivering data on admissions following cardiac surgery are a subset of the 33 hospitals delivering data on other admissions. We provide details of the numbers of admissions excluded. Of the nine hospitals supplying admissions following cardiac surgery, three (33.3 %) were academic and six (66.7 %) were teaching hospitals. Of the 33 hospitals providing data on admissions for reasons other than cardiac surgery, 3 (9.1 %) were academic, 17 (51.5 %) were teaching and 13 (39.4 %) were general hospitals.

The numbers and percentages of ICU admissions, the ICU treatment days and the median length of stay (LOS) for admissions following cardiac surgery and admissions for other reasons, attributable to the very elderly, in each calendar year between 2005 and 2014 are presented in Tables 2 and 3. The number of admissions following cardiac surgery attributable to the very elderly rose from 494 in 2005 to 909 in 2014 (*p* value = 0.0004), while the percentage of admissions attributable to this group rose from 6.7 % (95 % CI 6.2 % to 7.3 %) in 2005 to 11.0 % (95 % CI 10.3 % to 11.6 %) in 2014 (*p* value <0.0001). The number of treatment days attributable to the very elderly rose from 1,143 in 2005 to 1,843 in 2014 (*p* value = 0.0403) and the percentage of treatment days attributable to this group rose from 8.6 % (95 % CI 8.1 % to 9.0 %) in 2005 to 11.7 % (95 % CI 11.2 % to 12.2 %) in 2014 (*p* value = 0.0157). The number of admissions for reasons other than cardiac surgery that were attributable to the very elderly rose from 3,033 in 2005 to 4,952 in 2014 (*p* value <0.0001), while the percentage of admissions attributable to this group remained stable at 13.8 % (95 % CI 13.7 % to 13.9 %, *p* value = 0.1315). The number of treatment days attributable to the very elderly rose from 11,810 in 2005 to 15,234 in 2014 (*p* value = 0.0002), but the percentage of treatment days attributable to this group remained stable at 12.0 % (95 % CI 11.9 % to 12.0 %, *p* value = 0.1429). The number of other admissions attributable to the very elderly that fulfilled both the APACHE II and SAPS II mortality prediction model inclusion criteria and their average predicted probability of mortality are presented in Table 3. The APACHE II-predicted probability of mortality remained stable at 0.2950 (95 % CI 0.2989 to 0.2919, *p* value = 0.8563). The SAPS II-predicted probability of mortality remained stable at 0.3204 (95 % CI 0.3165 to 0.3243, *p* value = 0.3880). In addition, we present the number and percentage of admissions attributable to the very elderly for medical reasons and several co-morbidities in Table 3. The percentage of admissions, in which the patient was admitted for medical reasons, rose from 44.1 % (95 % CI 42.1 % to 46.2) to 55.3 % (53.7 % to 56.8 %, *p* value <0.0001). The percentage with chronic renal insufficiency rose from 5.0 % (95 % CI 4.2 % to 6.0 %) to 11.1 % (95 % CI 10.2 % to 12.1 %, *p* value <0.0001). The percentage with immunological insufficiency rose from 2.8 % (95 % CI 2.2 % to 3.6 %) to 6.5 % (95 % CI 5.8 % to 7.3 %, *p* value <0.0001). The percentage with a malignancy rose from 3.5 % (95 % CI 2.8 % to 4.3 %) to 5.9 % (95 % CI 5.2 % to 6.6 %, *p* value = 0.0062). The percentage with cardiovascular insufficiency remained stable at 7.9 % (95 % CI 7.6 % to 8.2 %, *p* value = 0.2456). The median ICU LOS remained stable at 1.61 days (interquartile range 0.85 to 3.68, *p* value = 0.3200).

**Table 2:** Numbers and percentages of ICU admissions following cardiac surgery and for other reasons, attributable to the very elderly in the period 2005 to 2014

Reason for ICU admission	Year	Total number of ICU admissions	Number (and percentage) of ICU admission attributable to the very elderly	Total number of ICU treatment days	Number (and percentage) of ICU treatment days attributable to the very elderly
Following cardiac surgery	2005	7,364	494 (6.7 %)	13,328	1,143 (8.6 %)
	2006	7,208	504 (7.0 %)	13,818	1,238 (9.0 %)
	2007	8,337	575 (6.9 %)	17,336	1,598 (9.2 %)
	2008	8,444	685 (8.1 %)	17,649	2,273 (12.9 %)
	2009	8,824	808 (9.2 %)	18,451	2,301 (12.5 %)
	2010	9,007	924 (10.3 %)	17,635	2,249 (12.8 %)
	2011	9,114	999 (11.0 %)	17,627	2,311 (13.1 %)
	2012	8,827	936 (10.6 %)	17,243	2,140 (12.4 %)
	2013	8,351	885 (10.6 %)	16,175	1,927 (11.9 %)
	2014	8,293	909 (11.0 %)	15,725	1,843 (11.7 %)
Range for hospitals^a^		593 to 1,579	71 to 173 (7.1 to 14.9 %)	732 to 3,034	114 to 375 (5.9 to 18.6 %)
Other reasons	2005	22,688	3,033 (13.4 %)	99,492	11,810 (11.9 %)
	2006	24,506	3,172 (12.9 %)	108,464	11,861 (10.9 %)
	2007	24,266	3,270 (13.5 %)	103,742	11,826 (11.4 %)
	2008	24,954	3,432 (13.8 %)	105,367	13,027 (12.4 %)
	2009	28,275	4,019 (14.2 %)	113,809	13,826 (12.1 %)
	2010	29,581	4,272 (14.4 %)	117,756	15,368 (13.1 %)
	2011	30,482	4,280 (14.0 %)	117,037	13,942 (11.9 %)
	2012	32,346	4,515 (14.0 %)	121,255	14,939 (12.3 %)
	2013	33,443	4,613 (13.8 %)	125,224	14,180 (11.3 %)
	2014	35,749	4,952 (13.9 %)	124,260	15,234 (12.3 %)
Range for hospitals^a^		329 to 2,349	32 to 350 (4.4 to 26.4 %)	1,194 to 8,078	75 to 902 (5.2 to 29.9 %)

^a^Data from 2014

**Table 3:** APACHE II- and SAPS II-predicated probability of mortality, proportion with chronic conditions and of those admitted for medical reasons and length of ICU stay for the very elderly

Year	Number of admissions^a^	Mean APACHE II-predicted probability of mortality	Mean SAPS II-predicted probability of mortality	Renal insufficiency	Cardiovascular insufficiency	Malignancy	Immunological insufficiency	Admissions for medical reasons	Median (interquartile range) length of ICU stay in days
2005	2,318	0.2786	0.2863	117 (5.0 %)	213 (9.2 %)	80 (3.5 %)	66 (2.8 %)	1,023 (44.1 %)	1.72 (0.88 to 4.02)
2006	2,542	0.2815	0.2976	134 (5.3 %)	196 (7.7 %)	112 (4.4 %)	73 (2.9 %)	1,097 (43.2 %)	1.63 (0.85 to 3.84)
2007	2,554	0.2853	0.3058	161 (6.3 %)	193 (7.6 %)	124 (4.9 %)	94 (3.7 %)	1,142 (44.7 %)	1.65 (0.86 to 3.81)
2008	2,763	0.3194	0.3421	232 (8.4 %)	212 (7.7 %)	132 (4.8 %)	140 (5.1 %)	1,321 (47.8 %)	1.75 (0.86 to 4.00)
2009	3,226	0.3013	0.3279	271 (8.4 %)	245 (7.6 %)	155 (4.8 %)	145 (4.5 %)	1,660 (51.5 %)	1.63 (0.85 to 3.66)
2010	3,408	0.3056	0.3277	345 (10.1 %)	308 (9.0 %)	190 (5.6 %)	160 (4.7 %)	1,767 (51.8 %)	1.66 (0.86 to 3.83)
2011	3,466	0.3107	0.3443	373 (10.8 %)	291 (8.4 %)	218 (6.3 %)	190 (5.5 %)	1,794 (51.8 %)	1.57 (0.84 to 3.64)
2012	3,784	0.2929	0.3136	383 (10.1 %)	285 (7.5 %)	174 (4.6 %)	192 (5.1 %)	2,048 (54.1 %)	1.59 (0.84 to 3.50)
2013	3,892	0.2853	0.3057	374 (9.6 %)	283 (7.3 %)	203 (5.2 %)	223 (5.7 %)	2,160 (55.5 %)	1.56 (0.84 to 3.32)
2014	4,085	0.2715	0.2857	455 (11.1 %)	313 (7.7 %)	240 (5.9 %)	265 (6.5 %)	2,257 (55.3 %)	1.47 (0.85 to 3.11)
Range for hospitals^b^	27 to 294	0.1037 to 0.4402	0.1068 to 0.4860	0 to 45 (0.0 % to 25.7 %)	0 to 34 (0.0 % to 33.8 %)	2 to 14 (2.0 % to 22.2 %)	0 to 26 (0.0 % to 22.2 %)	7 to 178 (23.3 % to 77.9 %)	0.89 to 2.83

^a^Admissions fulfilling both the acute physiology and chronic health evaluation II (APACHE II) and the simplified acute physiology score II (SAPS II) inclusion criteria. ^b^Data from 2014

## Discussion

In this study, we have examined changes in the percentage of hospital and ICU admissions in the Netherlands attributable to the very elderly in the period 2005 to 2014, and compared them with changes in the proportion of very elderly in the population as a whole. As the proportion of very elderly in the Dutch population increased, so did the percentage of hospital admissions. However, the percentage of, ICU admissions and treatment days attributable to the very elderly remained stable, except for ICU admissions and treatment days following cardiac surgery, which significantly increased from 2005 to 2014.. The severity of illness of very elderly patients, expressed by the APACHE II- and SAPS II-predicted probability of mortality, remained stable over time. However, the percentage of medical admissions and admissions of patients with chronic renal insufficiency, immunological insufficiency or a malignancy increased.

Our study confirms that the demographic changes occurring in Europe and in other high-income countries are also occurring in the Netherland. These changes have resulted in significant increases in the percentage of adults who are very elderly and percentage of hospital admissions attributable to this group. The percentage of ICU admissions attributable to the very elderly was similar to those reported in studies conducted in Australia and New Zealand [15], Denmark [22], France [17, 18], Italy [19], Norway [20] and Spain [21] and slightly higher than in Finland [16]. However, our finding that with the exception of admissions following cardiac surgery, the percentage of ICU admissions attributable to the very elderly did not increase between 2004 and 2013, contrasts with previous publications [15, 22]. In Australia and New Zealand, the ICU admission rates of very elderly patients increased by 5.6 % per year between 2000 and 2005 and in Denmark the percentage the percentage of ICU patients who were very elderly rose from 11.7 % in 2005 to 13.8 % in 2011. In our study, we only observed an increase in this percentage for cardiac surgery admissions, rising from 6.7 % in 2005 to 11.0 % in 2014. Previously, researchers ascribed observed increases to demographic changes and the introduction of new technologies and pharmaceutical agents [8, 15, 41, 42]. In Denmark, the percentage of ICU admissions attributable to the very elderly increased, while there was a decrease in absolute number of ICU admissions, and no change in the percentage of very elderly in the general population during the study period . This suggests that the increase of ICU admissions is primarily due to a change in admission policy in Denmark with regard to age. In contrast, in our study there was a significant increase in the percentage of very elderly adults in the general population (from 4.5 % in 2005 to 5,4 % in 2014) and this percentage was higher than in Denmark (4.1 % in both 2005 and 2011). In addition, the proportion of ICU admissions attributable to the very elderly admitted to the ICU in the Netherlands in 2005 was similar to the percentage in Denmark in 2011. Finally, our study cohort included more admissions with a longer observation period than compared to the study performed in Denmark.

Our finding that the percentage of Dutch ICU admissions attributable to the very elderly did not increase except for cardiac surgery patients could be explained by more strict ICU admission policies, due to changing opinions about treatment of the very elderly or experiences of poorer outcomes for the very elderly following ICU admission. Proactive treatment restrictions set on the wards on hospital admission in consultation with patients and relatives, such as do-not-resuscitate orders, and absence of ICU admission policies could have influenced our findings. Whether this policy is justified remains questionable as researchers have shown that elderly patients are more frequently rejected by the ICU than younger patients [43]. They also showed that elderly patients have higher mortality when admitted, but that the mortality benefit of ICU admission appears greater for elderly patients than for younger patients. Although limited healthcare resources may have negatively influenced decisions to admit very elderly patients to ICUs, this is not likely, because the absolute number of ICU admissions has increased for both younger and very elderly patients.

In contrast, we demonstrated that the increase in the percentages of admissions following cardiac surgery that are attributable to the very elderly is larger than the increase in the percentage of hospital admissions attributable to the very elderly. This may be due to improvements in techniques in cardiac surgery and general medical care, and changed ethical reasoning around cardiac surgery in elderly patients. This finding is consistent with an earlier Dutch report, which mentioned increases in the mean age of patients undergoing cardiothoracic surgery and in the proportion of patients aged 76 years and older between 1995 and 2011 [34].

Decrease in absolute number of hospital admissions after 2012 is most probably due to exclusion of day-case admissions from the hospital admissions. Since 2013 health insurance companies have forced hospitals to register day-case admissions as outpatient cases for financial payout. These changes were partly implemented by hospitals in 2013 and complete implementation was only reached in 2014.

The strengths of our study include the combination of data from the three registries (Statistics Netherlands, Dutch Hospital data and the NICE registry), the long study period and the large number of admissions included. We included hospitals that provided data to the NICE registry for the whole period from 2005 to 2014 to investigate long-term trends. As hospitals voluntarily participate in the NICE registry, it is reasonable to assume that missing data caused by a hospital not choosing to participate are non-ignorable [36]. Therefore, we decided to focus on hospitals that provided data to the NICE registry for the whole period from 2005 to 2014 to investigate long-term trends and exclude a bias caused by differences in patient populations between participating hospitals.

However, our study also has some limitations. First, the changes in the percentage of admissions attributable to the very elderly in individual hospitals may be substantially different from the group trends. Second, we modeled the NICE data using mixed-effects logistic models, but modeled the Statistics Netherlands data and Dutch Hospital Data using ordinary logistic models. This is because we assumed that the Statistics Netherlands data and Dutch Hospital Data had total population coverage, whereas we obtained NICE data from a selection of hospitals covering only part of the population of ICU admissions in the Netherlands. Third, the data presented in this paper reflect the first ICU admissions within any hospital stay. Hence, the total ICU admissions of the very elderly, including re-admissions, may be higher than suggested by our results if the very elderly have significantly more ICU readmissions within single hospital admissions. However, this is not likely because the very elderly are more likely than younger patients to have no-return or do-not-resuscitate orders. Fourth, the burden of the very elderly could only be expressed by the number of treatment days because the NICE registry does not currently contain data on nursing workload or end-of-life decisions. Fifth, we used the older APACHE II and SAPS II models to calculate the predicted probability of mortality, as data for the APACHE IV model was only available in the NICE registry from 2008. Sixth, at the point of data extraction, registration of hospital admissions at the Dutch Hospital Data registry was not complete for 2014. However, admissions of almost 96 % of the hospitals had already been registered for this year. In addition, the absolute number of hospital admissions has decreased since 2012 due to a change in the way health insurance companies reimbursed day-case admissions. These changes were partly implemented in 2013 and completely implemented in 2014.

Our results are of importance for the Netherlands, but possibly also for other European countries with comparable healthcare systems. In the past, Dutch researchers predicted that, as the population ages, the pressure on healthcare facilities, including ICUs, would continue to increase. They expected that between 2006 and 2021, ICU treatment days for the very elderly would increase by 32 % [12]. We did find a significant rise in the number of treatment days for the very elderly. However, the percentage of ICU treatment days attributable to this group remained stable meaning that the increase in ICU admissions and treatment days are equally distributed over younger and very elderly patients. This finding does not support statements made by researchers suggesting that the increasing percentage of very elderly in the population in combination with developments in healthcare technology would fuel ICU admissions of the very elderly. Based on our results, we question whether these massive increases will occur. Our models have not been constructed to predict demand for healthcare resources, but could still be useful when modelling future ICU capacity requirements in the Netherlands and in countries with similar patterns of population aging.

However, to make rational decisions about the admission of the very elderly to the ICU, it is important to have data on short-term and long-term outcomes, including quality of life after discharge of the hospital. Intensive care specialists need to know whether the very elderly benefit from ICU admission as much as younger patients, whether they have an acceptable quality of life after ICU treatment and which patient characteristics predict good outcomes. Future research should examine these topics to enable optimal allocation of ICU resources and guide ethical decisions as to whether the very elderly should be offered ICU care.

## Conclusion

Although the Dutch population is aging and both the absolute number and percentage of hospital admissions attributable to the very elderly increased between 2005 and 2014, we did not see an increase in the percentage of general ICU admissions and treatment days attributable to the very elderly non-cardiac surgery patients in this period. In contrast, we have shown that the percentage of ICU admissions and treatment days following cardiac surgery that are attributable to the very elderly increased between 2005 and 2014.

## Key messages

- The Statistics Netherlands data show that the percentage of the very elderly in the Netherlands, increased from 4.5 % in 2005 to 5.4 % in 2014 (*p* value <0.0001)
- This aging of the Dutch population resulted in an increase in the percentage of very elderly admitted to Dutch hospitals from 9.0 % in 2005 to 10.6 % in 2014 (*p* value <0.0001)
- We demonstrated that the percentage of ICU admissions attributable to very elderly was stable at 14 % between 2005 and 2014, with the exception of admissions following cardiac surgery, for which the percentage rose from 6.7 % in 2005 to 11 % in 2014
- The number of ICU treatment days increased but the percentage of ICU treatment days attributable to the very elderly remained stable, except for admissions following cardiac surgery
- The severity of illness of very elderly patients admitted to the ICU remained stable

## Additional file

Additional file 1:
**Acute physiology and chronic health evaluation II (APACHE II) and APACHE IV reasons for intensive care unit admission related to cardiac surgery. **(PDF 170 kb)

## Abbreviations

APACHE: acute physiology and chronic health evaluation
CI: confidence interval
ICU: intensive care unit
LOS: length of stay
NICE: Dutch National Intensive Care Evaluation
SAPS: simplified acute physiology score
