# Supplementary material for: Trends in hospital and intensive care admissions in the Netherlands attributable to the very elderly in an ageing population
Source: Crit Care. 2015 Sep 30;19:353. doi: 10.1186/s13054-015-1061-z (PMC4588268; doi:10.1186/s13054-015-1061-z)
Supplement: Additional file 1: — Acute physiology and chronic health evaluation II (APACHE II) and APACHE IV reasons for intensive care unit admission related to cardiac surgery. (PDF 170 kb) [file 13054_2015_1061_MOESM1_ESM.pdf]

Additional file 1

APACHE II and APACHE IV reasons for intensive care unit admission related to cardiac surgery.

	**Reasons for intensive care unit admission**
**APACHE II**	Heart valve surgery - surgical
	Chronic cardiovascular disease - surgical
**APACHE IV**	Aortic and mitral valve replacement
	Aortic valve replacement, isolated
	Atrial septal defect repair
	Coronary artery bypass grafting alone
	Coronary artery bypass grafting redo
	Coronary artery bypass grafting redo with other operation
	Coronary artery bypass grafting redo with valve repair/replacement
	Coronary artery bypass grafting with aortic valve replacement
	Coronary artery bypass grafting with double valve repair/replacement
	Coronary artery bypass grafting with mitral valve repair
	Coronary artery bypass grafting with mitral valve replacement
	Coronary artery bypass grafting with other operation
	Coronary artery bypass grafting with pulmonic or tricuspid valve repair or replacement only
	Coronary artery bypass grafting, minimally invasive, mid-CABG
	Mitral valve repair
	Mitral valve replacement
	Tricuspid valve surgery
	Ventricular septal defect repair
